# Systematic review and meta-analysis: association between obesity/overweight and surgical complications in IBD

**DOI:** 10.1007/s00384-022-04190-y

**Published:** 2022-05-31

**Authors:** Ke Jiang, Bangsheng Chen, Dandi Lou, Mengting Zhang, Yetan Shi, Wei Dai, Jingyi Shen, Bin Zhou, Jinxing Hu

**Affiliations:** 1 The Second Clinical Medical College, Zhejiang Chinese Medical University, Hangzhou, Zhejiang China; 2 Emergency Medical Center, Ningbo Yinzhou No. 2 Hospital, Ningbo, Zhejiang China; 3 The First Clinical Medical College, Zhejiang Chinese Medical University, Hangzhou, Zhejiang China; 4 Department of General Surgery, HwaMei Hospital, University of Chinese Academy of Sciences, Ningbo, Zhejiang China; 5 Department of Endocrinology, HwaMei Hospital, University of Chinese Academy of Sciences, Haishu District, Northwest Street 41, Ningbo, 315010 Zhejiang China

**Keywords:** Obesity, Overweight, Inflammatory bowel disease, Complications, Meta-analysis

## Abstract

**Purpose:**

While the prevalence of obesity in inflammatory bowel disease (IBD) patients is rapidly increasing, it is unclear whether obesity affects surgical outcomes in this population. This meta-analysis aims to assess the impact of obesity/overweight on patients undergoing surgery for IBD.

**Methods:**

Databases (PubMed, Web of Science, Cochrane Library, and Springer) were searched through September 2021. The meta-analysis included patients with surgically treated IBD to investigate the impact of obesity/overweight on this population. Primary outcomes included overall complications, infectious complications, noninfectious complications, and conversion to laparotomy.

**Results:**

Fifteen studies totaling 12,622 IBD patients were enrolled. Compared with nonobese (including overweight) patients, obese IBD patients have increased the risk in terms of overall complications (OR = 1.45, *p* < 0.001), infectious complications (OR = 1.48, *p* = 0.003) (especially wound complications), as well as conversion to laparotomy (OR = 1.90, *p* < 0.001). Among the noninfectious complications, only the incidence of visceral injury (OR = 2.36, *p* = 0.05) had significantly increased. Compared with non-overweight patients, the risk of developing wound complications (OR = 1.65, *p* = 0.01) and sepsis (OR = 1.73, *p* = 0.007) were increased in overweight patients, but the rates of overall complications (OR = 1.04, *p* = 0.81), infectious complications (OR = 1.31, *p* = 0.07), and conversion to laparotomy (OR = 1.33, *p* = 0.08) associated with body mass index (BMI) were not significantly different.

**Conclusion:**

Obesity is a risk factor for surgical complications in IBD patients, mainly reflected in infectious complications. Moreover, obese patients seem to have a more common chance of developing surgical complications than overweight patients.

**Supplementary information:**

The online version contains supplementary material available at 10.1007/s00384-022-04190-y.

## Introduction

Previously, more than 15 million and 2 million people in North America and Europe were diagnosed with inflammatory bowel disease (IBD), respectively [1, 2]. According to epidemiological studies, with the continuous development of some newly industrialized countries (such as Asia and Africa), IBD has become a global disease in the twenty-first century [1, 3, 4]. Marasmus is usually considered an adverse factor in IBD patients. With the activation of the autoimmune system, tissue repair, and changes in drug-nutrition interactions, the nutritional needs of IBD patients have far exceeded those of the general population. Therefore, the loss of body weight tends to increase the risk of malnutrition [5], which in turn lifts the occurrence of postoperative complications [6, 7].

Studies have revealed that obesity can significantly increase the risk of perioperative complications in other general surgical procedures, including anastomotic leakage, wound infection, intestinal obstruction, and blood transfusion. In addition, it has been reported that, from a surgical perspective, obesity/overweight will increase the conversion rate from laparoscopic to open surgery in patients [8]. According to the earliest study based on the French population, obesity/overweight was uncommon in IBD patients, with only 3.6% of the 2065 CD patients being obese [9]. However, the incidence of obesity/overweight in IBD patients is increasing [10, 11]. In an observational study in Scotland [12], Steed and his colleagues found that 18% of the IBD population was obese (body mass index (BMI) ≥ 30 kg/m^2^) (total proportion of Scottish population approximately 22%) and 38% of IBD patients were overweight (BMI ≥ 25 kg/m^2^). At present, on the one hand, postoperative complications in patients with IBD are more common than those requiring surgery for other diseases, which may be related to adverse clinical factors related to surgery (such as immunosuppressive drugs) and factors that make surgery more challenging (such as intestinal wall fragility). On the other hand, due to the increase in the number of obese/overweight patients as a special type (usually we believe that IBD patients are mostly thin due to malnutrition) of IBD patients, the relationship between obesity/overweight and surgical complications in IBD patients has attracted more attention and controversy. According to an analysis by Causey et al. [13] and Abd EI Aziz et al. [14], obesity/overweight would increase the occurrence of perioperative complications in IBD patients. In contrast, other studies [15, 16] reported that surgical complications in obesity/overweight IBD patients were not significantly different from those in normal weight.

Despite the relationship between obese IBD patients and surgical complications has been explored [17], the existing meta-analysis contains not many articles and did not perform further analysis on overweight patients. Herein, by reviewing and collecting relevant studies, we try to investigate the relationship between obesity/overweight and surgical complications in IBD patients.

## Materials and methods

### Literature search strategy

This meta-analysis was conducted under the guidelines of the Preferred Reporting Items for Systematic Reviews and Meta-Analyses (PRISMA). From database establishment to November 2021, studies on the relationship between obesity/overweight and surgical complications in patients with IBD were retrieved. Data from different electronic databases (PubMed, Web of Science, Cochrane Library, and Springer Link) had been searched and extracted, which finally formed this meta-analysis. When it comes to the searching for the topic, the article used a combination of free-text terms and medical subject headings terms. The search terms used include: (Inflammatory bowel disease OR Crohn’s disease OR Ulcerative colitis OR IBD OR UC OR CD) AND (Obesity OR Obese OR BMI OR Fat OR Adiposity OR Body mass index OR Corpulence OR Overweight) AND (Postoperative OR Perioperative OR Surgery OR Operation) AND (Outcomes OR Complications OR Results). Furthermore, two researchers conducted a preliminary screening of the titles and abstracts of the retrieved articles independently. In order to comprehensively review potentially related studies, manual searching of references and citations of related articles were carried out.

### Inclusion and exclusion criteria

Studies that have met the following criteria were included: (1) The content of the article was related to the relationship between obesity/overweight and surgical complications. (2) Objectives of the study were for adults or children with IBD. (3) The study was observational (case–control or cohort study). (4) The exposed groups were obese (BMI ≥ 30 kg/m^2^) or overweight (25 kg/m^2^ ≤ BMI < 30 kg/m^2^) patients, and the objectives in the nonexposed groups were nonobese (including overweight) or non-overweight.

Studies that have met one of the following exclusion criteria were excluded: (1) No data on the association of obesity/overweight IBD patients with surgical complications were provided, or data could not be extracted. (2) In addition to IBD, patients had other health conditions (e.g., uncertainty colitis, familial polyposis (FAP), tumors). (3) The study was published not in English. (4) The original article had only an abstract published or was absent in the full text. (5) The study content could not be combined with the data of other articles.

When the article was updated repeatedly, the latest or the most complete research would be involved.

### Outcome measures

The result of this meta-analysis mainly focused on surgical complications in IBD patients undergoing various types of surgery. Patients were divided into two groups according to their BMI: patients with obesity (BMI ≥ 30 kg/m^2^) vs. patients without obesity (including overweight patients) (BMI < 30 kg/m^2^). Then for further analysis, patients were classified as overweight (25 kg/m^2^ ≤ BMI < 30 kg/m^2^) and non-overweight (BMI < 25 kg/m^2^). Complications included overall complications, infections complications (wound complications, sepsis, respiratory infection, etc.), noninfectious complications (ileus, visceral injury, bleeding, etc.), and conversion (adhesions, difficult exposure, etc.).

### Quality assessment and data extraction

The study data were independently extracted by two researchers, then reviewed and confirmed by a third investigator. We recorded information in author, year of publication, country of study, time period, patient type, quantity, BMI, follow-up period, surgery type, and postoperative complications. Furthermore, the included observational studies were evaluated using the Newcastle–Ottawa Quality Assessment Scale (NOS), which includes three major aspects: selection, comparability, and outcomes. Each study was assigned a score between 0 and 9. Articles ≥ 6 points were considered high-quality studies.

### Statistical analysis

The data were analyzed via Review Manager 5.3 analysis software. Heterogeneity between studies was assessed by using the *I*^2^ statistics. Due to differences in study design and detailed information about patients, this study used a random-effects model to analyze to improve credibility. Meanwhile, odds ratio (OR) and 95% confidence interval (95%Cl) were adopted in the results. OR > 1 was the indication of obesity/overweight patients being riskier to have postoperative complications than controls. On the contrary, OR < 1 was considered obesity/overweight, which could reduce the risk of postoperative complications. When *p* < 0.05, the result would be considered to have statistical significance. Funnel plots were used to test publication bias, while sensitivity analysis was used to evaluate the stability of the results.

## Results

### Study selection

**Fig. 1:**
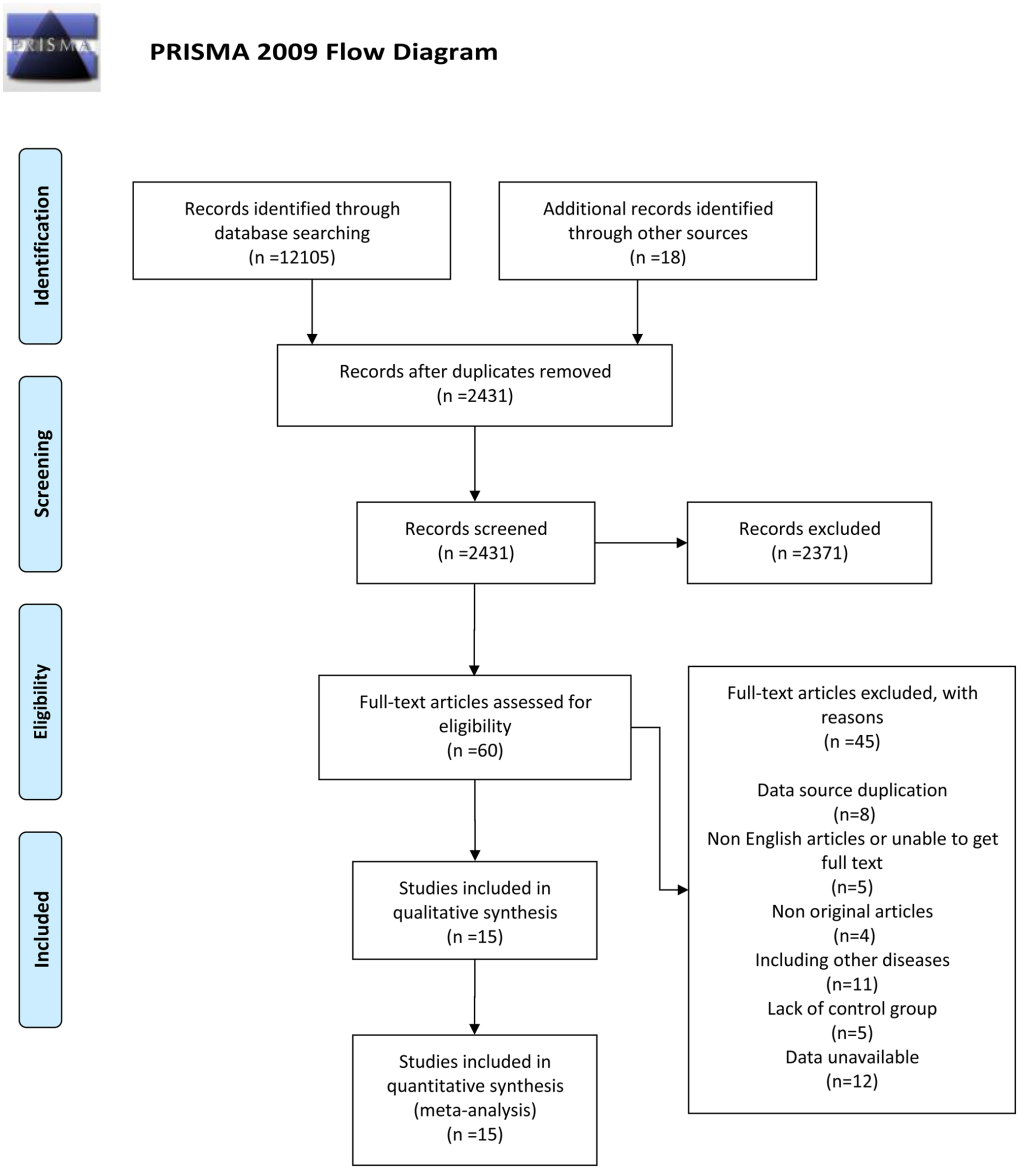
Flow diagram of selection

From the four electronic databases, we initially collected 12,105 studies that were closely related to the subject mentioned before. In addition, after manual retrieval, 18 studies were included. After preliminary screening and review, 2371 studies were excluded due to their inconsistency with the research topic, or the articles were review studies, or the type of surgery was inconsistent (such as weight loss surgery). Moreover, after carefully reading, reviewing, and confirming the full-text content, a total of 15 studies were finally included [10, 13–16, 18–27] to form this meta-analysis. The detailed inclusion and exclusion process of the articles has been shown in Fig. 1.

### Characteristics of the included studies

The characteristics of the included studies were listed in Table 1. From 2010 to 2021, a total of 15 studies were reported. All of them were studies in investigating the link between obese or overweight IBD patients and surgical complications, where 12,622 IBD patients (adults and children) were included. Among them, there were 2294 obese patients and at least 1119 overweight patients. All 15 included studies were retrospective observational studies. One of these 15 observational studies [14] used the propensity score-matched analysis to analyze the data. Besides, one was jointly completed by researchers from three countries: The Netherlands, Belgium, and the USA, two were from Japan [21, 25], and the remaining 12 were reported from the USA [10, 13–16, 18, 20, 22–24, 26, 27]. In this meta-analysis, the patients in one article were children only [26], and the rest were all adults. In addition, there were four studies with only CD patients [13, 18, 20, 27], and five studies with only UC patients [14, 21, 22, 24, 25]. All patients underwent different types of surgery. There were 14 studies [10, 13–16, 18, 19, 21–27] that followed up with the recruited patients for at least 30 days after surgery.

**Table 1:** Characteristics of included studies in the meta-analysis

**Author**	**Year**	**Country**	**Time period**	**Patients**	**Disease types**	**Study group**				**Follow-up period**	**Surgery type**
						Obese (≥ 30 kg/m^2^)	Nonobese (< 30 kg/m^2^)	Overweight (25–30 kg/m^2^)	Non-overweight (< 25 kg/m^2^)		
Canedo	2010	USA	2000–2008	Adult	IBD	NA	NA	86	127	60 days	Laparoscopic colorectal resection
Causey	2011	USA	2005–2008	Adult	CD	379	1940	NA	NA	30 days	Open colectomy Laparoscopic colectomy Small-bowel procedures Proctectomy Stoma-related procedures
Krane	2013	USA	2002–2011	Adult	IBD	85	541	206	335	6 months	Laparoscopic colorectal surgery
Stidham	2015	USA	2004–2011	Adult	CD	25	244	57	187	30 days	Intestinal resection
Sahami	2016	The Netherlands; Belgium; USA	1990–2015	Adult	IBD	48	538	124	414	35 months	IPAA
Guardado	2016	USA	2000–2014	Adult	IBD	65	326	105	221	30 days	Colorectal surgery
Manne	2015	USA	2000–2013	Adult	CD	16	102	32	70	NA	CDPF
Okita	2017	Japan	2002–2016	Adult	UC	NA	NA	76	129	93 ± 47 days	IPAA
McKenna	2017	USA	2002–2013	Adult	UC	154	755	NA	NA	30 days	IPAA
Heimann	2018	USA	1976–2014	Adult	IBD	90	910	207	703	8 years	Open-bowel resection
McKenna	2018	USA	2012–2015	Adult	UC	835	2566	NA	NA	30 days	IPAA
Horio	2018	Japan	2012–2015	Adult	UC	NA	NA	16	149	30 days	IPAA
Kao	2019	USA	2012–2015	Children	IBD	67	791	112	679	30 days	Colorectal surgery
McKenna	2019	USA	2007–2017	Adult	CD	128	630	178	452	30 days	Ileocolic resection
Abd EI Aziz	2021	USA	2007–2018	Adult	UC	402	402	NA	NA	30 days	MIS total proctocolectomy with IPAA

*IBD* inflammatory bowel disease, *UC* ulcerative colitis, *CD* Crohn’s disease, *NA* not available, *IPAA* ileal pouch-anal anastomosis, *MIS* minimal invasive, *ACS-NSQIP* American College of Surgeons National Surgical Quality Improvement Program, *COPF* CD-associated perianal fistula

### Quality assessment of the included studies

NOS was used to assess the quality of the 15 observational studies. Articles with a score of < 6 were considered lower quality. All 15 of the articles in this meta-study were graded with a score of ≥ 6, proving to be of higher quality. Detailed quality assessment results are shown in Supplementary Table 1.

### Analysis results of complications

**Fig. 2:**
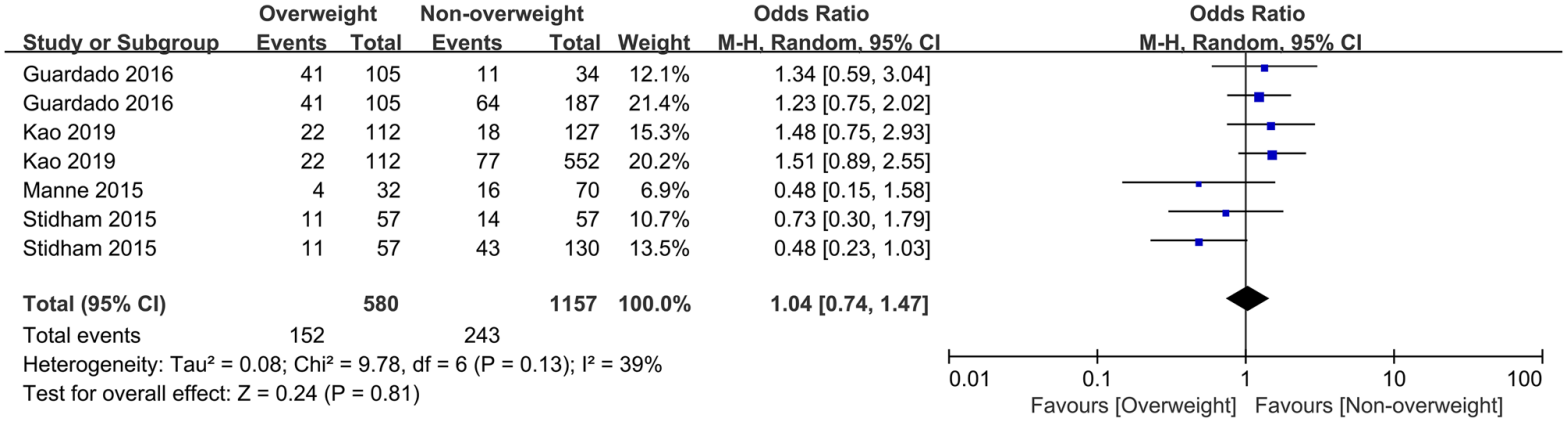
Forest plot of the association between overall complications and overweight

As shown in Fig. 2, compared with non-overweight IBD patients, complications in overweight IBD patients do not differ in overall complications (OR = 1.04, *p* = 0.81). Further subgroup analysis of surgical complications to compare overweight and non-overweight IBD patients with normal illustrates that overweight patients have a higher risk of infection in terms of overall infection complications (OR = 1.31, *p* = 0.07) without statistical significance. Additionally, being overweight increases the risk of experiencing wound complications (OR = 1.65, *p* = 0.01), mainly incisional hernia (IH)/fascial dehiscence (OR = 1.62, *p* = 0.002). At the same time, being overweight also increases the risk of postoperative sepsis (OR = 1.73, *p* = 0.007). However, according to the included studies, being overweight does not increase the incidence of superficial surgical site infection (sSSI), respiratory infection, urinary tract infection (UTI), abscess, or anastomotic leak.

In terms of noninfectious complications, the risk of included complications (ileus, visceral injury, venous thromboembolism (VTE), bleeding, and return to the operating room) do not differ between the exposed groups compared and the non-exposure group. Apart from this, the univariate analysis shows that being overweight increases the risk of conversion (OR = 1.33, *p* = 0.08) without statistical significance. Additional studies on the reasons for conversion were conducted, and the results were divided into four categories, including adhesions, bleeding, inflammatory mass, and difficult exposure. However, none of them show that being overweight promotes the occurrence of the above complications. See Table 2 for details.

**Table 2:** Total postoperative complications for overweight vs. non-overweight

**Overweight vs. non-overweight**	**No. of studies**	**Participants**	**OR**	**95%CI**	***p***	**Heterogeneity** **(*I***^**2**^**) (%)**
Infections	2	956	1.31	0.97–1.77	0.07	0
Wound complications	3	1330	1.65	1.13–2.42	0.01	0
sSSI	2	1421	1.10	0.36–3.39	0.87	46
IH/fascial dehiscence	3	1775	1.62	1.20–2.19	0.002	0
Respiratory infection	3	1747	2.00	0.65–6.15	0.23	56
UTI	3	1747	1.31	0.64–2.69	0.46	37
Sepsis	3	1634	1.73	1.16–2.57	0.007	0
Abscess	2	539	0.53	0.19–1.52	0.24	0
Anastomotic leak	4	1242	1.67	0.88–3.15	0.12	33
Noninfections						
Ileus	3	1169	0.97	0.64–1.48	0.89	1
VTE	2	956	1.19	0.50–2.84	0.70	0
Visceral injury	2	867	0.90	0.28–2.86	0.85	0
Bleeding	3	1282	2.04	0.95–4.36	0.07	30
Return to the operating room	4	2286	1.34	0.97–1.85	0.08	0
Conversion	4	1710	1.33	0.97–1.81	0.08	0
Adhesions	3	1080	0.91	0.49–1.71	0.78	0
Bleeding	3	1080	1.78	0.39–8.16	0.46	0
Inflammatory mass	2	867	1.87	0.75–4.70	0.18	0
Difficult exposure	2	867	1.01	0.27–3.76	0.99	0

*OR* odds ratio, *CI* confidence interval, *sSSI* superficial surgical site infection, *IH* incisional hernia, *UTI* urinary tract infection, *VTE* venous thromboembolism

**Fig. 3:**
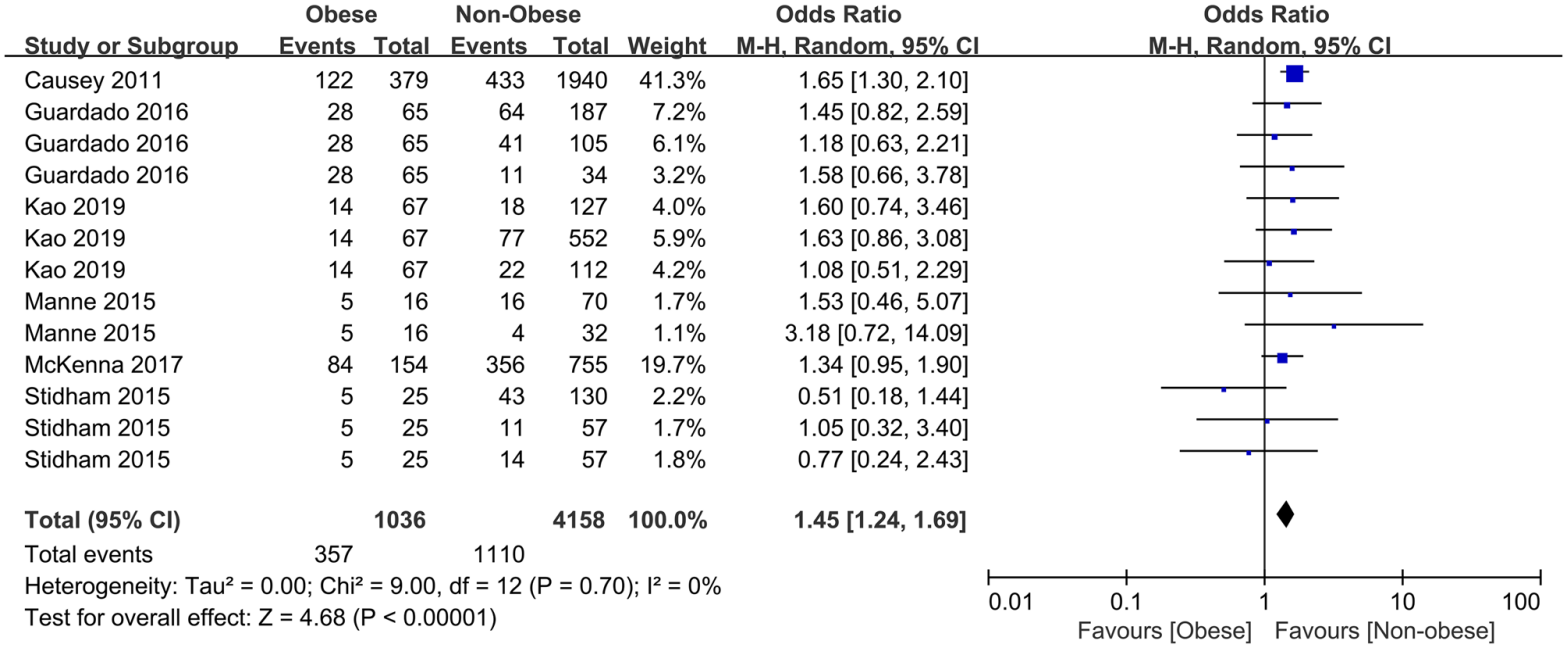
Forest plot of the association between overall complications and obesity

At the same time, studies based on the obese and nonobese (including overweight) IBD patients are also conducted. According to the research results in Fig. 3, obesity is a risk factor for the development of any complications (OR = 1.45, *p* < 0.001). The effects of being obese and nonobese on specific postoperative complications are further analyzed. Specific contents reference Table 3. Obesity increases the risk of complications in infections (OR = 1.48, *p* = 0.003). In addition, the risk of wound complications (OR = 1.81, *p* < 0.001) and UTI (OR = 1.37, *p* = 0.03) are also increased. Especially in wound complications, compared with nonobese IBD patients, obese patients are riskier of experiencing deep surgical site infection (dSSI) (OR = 2.05, *p* = 0.06), surgical site infection (SSI), sSSI, organ/space SSI, and IH//fascial dehiscence, but the difference does not reach statistical significance in dSSI. Furthermore, the risk of other infectious complications (septic shock, abscess, anastomotic leak, etc.) shows no difference.

**Table 3:** Total postoperative complications for obese vs. nonobese

**Obese vs. nonobese**	**No. of studies**	**Participants**	**OR**	**95%CI**	***p***	**Heterogeneity** **(*I***^**2**^**) (%)**
Infections	2	1149	1.48	1.15–1.91	0.003	0
Wound complications	5	5281	1.81	1.38–2.37	< 0.001	0
SSI	2	1662	1.53	1.12–2.08	0.007	0
sSSI	4	4739	1.91	1.13–3.22	0.02	62
dSSI	3	3981	2.05	0.96–4.37	0.06	40
Organ/space SSI	3	3981	1.48	1.09–2.00	0.01	0
IH//fascial dehiscence	3	2015	1.48	1.00–2.19	0.05	24
Respiratory infection	6	6039	1.33	0.87–2.03	0.19	0
UTI	6	6039	1.37	1.04–1.82	0.03	0
Sepsis	5	5648	0.72	0.46–1.13	0.15	53
Septic shock	3	3981	1.76	0.68–4.57	0.24	24
Abscess	2	1300	1.97	0.96–4.02	0.06	0
Anastomotic leak	2	977	1.49	0.93–2.40	0.10	0
Noninfections						
Ileus	3	2058	1.15	0.83–1.58	0.40	0
VTE	4	2862	0.96	0.55–1.65	0.88	0
MACE	3	3514	0.65	0.20–2.15	0.48	0
Unplanned intubation	2	3177	1.85	0.64–5.35	0.26	12
Visceral injury	2	1017	2.36	1.01–5.54	0.05	0
Urinary retention	2	1300	1.49	0.88–2.53	0.14	0
Renal complications	3	3514	1.10	0.49–2.47	0.81	0
Bleeding	2	1249	1.07	0.58–1.95	0.83	0
Blood transfusion	3	4032	1.44	0.97–2.12	0.07	0
Death	3	4032	1.02	0.25–4.15	0.98	0
Readmission	2	4159	1.07	0.90–1.27	0.45	0
Return to the operating room	4	2631	1.08	0.79–1.48	0.62	0
Conversion	3	1775	1.90	1.43–2.52	< 0.001	0
Adhesions	2	1017	0.51	0.22–1.16	0.11	0
Bleeding	2	1017	1.79	0.53–5.98	0.35	0
Inflammatory mass	2	1017	1.39	0.60–3.26	0.44	0
Difficult exposure	2	1017	2.77	1.07–7.21	0.04	0

*OR* odds ratio, *CI* confidence interval, *SSI* surgical site infection, *sSSI* superficial surgical site infection, *dSSI* deep surgical site infection, *IH* incisional hernia, *UTI* urinary tract infection, *VTE* venous thromboembolism, *MACE* major adverse cardiovascular events

Among the noninfectious complications, only the risk of visceral injury (OR = 2.36, *p* = 0.05) is increased. No difference between other included complications, such as urinary retention, renal complications, and bleeding is statistically significant. According to the three studies, the increase in conversion (OR = 1.90, *p* < 0.001) rate is closely related to obesity, which is mainly due to the difficulty in exposure (OR = 2.77, *p* = 0.04) during surgery.

### Sensitivity analysis and publication bias

Funnel plots (Figs. S1 and S2) were applied to test the relationship between obesity/overweight and overall complications. It is revealed that the funnel plot associated with overweight and overall complications is roughly symmetrical (see Fig. S1 for details), indicating that there is no significant publication bias. The result is stable after removing the studies one by one. In addition, the forest plot of the association between obesity and overall complications is also tested for publication bias and shows symmetry overall (see Fig. S2 for details). The result also remains stable after removing the studies one by one.

## Discussion

Through systematic reviews and meta-analyses of the included studies, the relationship between obesity/overweight and surgical complications in patients with IBD is investigated. The findings suggest that obesity/overweight obviously influences surgical outcomes. Compared with non-overweight patients with IBD, those overweight are at increased risk of experiencing wound complications, IH/fasciothesis, and sepsis. However, no significant difference in overall postoperative complications is observed. Whether obesity plays a role is also investigated. Compared with nonobese (including overweight) IBD patients, obesity increases the incidence of overall postoperative complications, infectious complications (especially wound complications), visceral injury, and conversion to open surgery. Furthermore, obesity or overweight does not increase the risk of other complications included in this meta-analysis, such as an anastomotic leak, abscess, VTE, ileus, and blood transfusion.

The occurrence of overall complications in IBD patients is strongly associated with being obese, but not with being overweight. This may be related to the higher BMI in obese patients. Causey et al. [13] found a significant increase in the overall complication rate with increasing BMI with an almost linear correlation between them. This suggests that even small weight changes could affect the outcome and that the incidence of overall complications differs only when the patient’s BMI breaks a threshold. Canedo et al. [15] study has shown that overweight IBD patients are not riskier in undergoing laparoscopic bowel resection compared to normal. This could be because the BMI of overweight patients does not reach the threshold. Moreover, it has shown that overweight patients have nutritional reserves and an efficient metabolic state to be better prepared for surgery [28]. Obesity has a large interval range for BMI, and it can be divided into three levels: obesity I (30 kg/m^2^ < BMI ≤ 35 kg/m^2^), obesity II (35 kg/m^2^ < BMI ≤ 40 kg/m^2^), and obesity III (BMI > 40 kg/m^2^) [29]. Although it has been experimentally stated that some patients with mild obesity have reduced comorbidities, the study also showed that grade III obesity was significantly associated with an increased incidence of postoperative complications [29]. As a result, obesity has long been recognized as a potential risk factor for poor outcomes in various surgical procedures [30]. The number of studies related to anesthesia [31], gastroenterology [32], and plastic surgery [33] describe increased morbidity and mortality associated with surgery in obese people. This may be because obese patients have an increased risk of accompanying several diseases such as diabetes, hypertension, renal impairment, and atherosclerotic vascular disease [34–36]. Similarly, obesity may also contribute to these conditions in IBD patients so that they increase the risk of overall surgical complications.

Further analysis of postoperative complications is performed. In terms of infectious complications, overweight IBD patients are not riskier to experience infectious complications. However, obesity increases the risk of infectious complications in IBD patients. The studies by [10, 14, 22, 26] have produced consistent results with the mentioned observation that patients with IBD were at increased risk of having infectious complications, particularly wound complications with increasing BMI. Similar conclusions were also drawn in obese patients with other diseases by Wahl et al. [37]. Obesity has an important impact on immune function and homeostasis [38]. It is described as a state of systemic inflammation with C-reactive protein (CRP) levels elevating in the absence of inflammatory and infectious etiologies in obese patients [11, 39, 40]. This may be mediated by cytokines (e.g., interleukin 6, tumor necrosis factor-α), neuropeptides (e.g., substance P), as well as recently identified adipokines (e.g., adiponectin, resistin) [11, 39]. These molecules can be produced in adipocytes or macrophages and lymphocytes infiltrating mesenteric fat. Mesenteric fat of patients with active IBD overexpresses [11, 41] cytokines, and the overexpression correlates with adipocyte mass [11, 42]. Substance P has been shown to play a proinflammatory role in obesity and IBD. This neuropeptide has a direct effect on adipose tissue expansion while creating a proinflammatory milieu [43]. In addition, adipocytokines are involved in inflammatory and metabolic pathways. Preliminary results on the overexpression of adipocytokines such as adiponectin and resistin in mesenteric adipose tissue of CD patients suggest that adipocytokines may play an important role in the pathogenesis of CD. The increased inflammatory response exacerbates the possibility of infection. However, this may be due to the extent of being overweight does not reach the threshold mentioned above, and only obese IBD patients have an increased risk in infectious complications. The report by Guardado et al. [16] contradicts our results. They believed that there is no difference in the incidence of postoperative infection complications or wound infection in obese IBD patients. This may be related to the different surgical methods. In their study, most patients underwent laparoscopic surgery, which may be beneficial to improve postoperative pain, reduce incision size, and reduce the inflammatory response.

On the other hand, overweight patients have an increased risk of wound complications, and obese patients have an increased incidence of SSI and wound complications. This may be related to the presence of relatively avascular adipose tissue mass, the increase of local trauma caused by abdominal wall contraction, the decrease of wound oxygen tension, the decrease in antibiotic penetration/concentration, and the decrease in the immune system and anti-infection ability in the state of overall inflammation [30, 37, 44–47]. On the other hand, the timing of surgical intervention is also very critical. The length of the disease course can affect the postoperative outcome. Patients with a disease course are usually more severely ill than those with a milder disease course, which makes them more susceptible to infection, resulting in a worse prognosis [48]. The effects of other infectious factors, such as ischemia along the suture line, large wound area, and insufficient collagen synthesis in IBD patients are also not negligible [32].

Current pharmacological treatment for IBD mainly includes 5-aminosalicylate, corticosteroids, anti-TNF (tumor necrosis factor) drugs (infliximab (IFX), adalimumab (ADA)), anti-integrin preparation (vedolizumab (VDZ)) [49–53], all of which may reduce the occurrence of infectious complications to some extent. Since the pharmacokinetics of the medications in obese patients are not the same as that in the normal, the efficacy of these drugs may be altered in obese patients. According to existing reports, high body weight has been identified as a risk factor associated with increased drug clearance, which leads to shortened half-life and lower drug concentrations. For example, both early losses of response to IFX and an increase in dose during ADA therapy are related to the increase in BMI [54, 55]. This effect may be related to rapid proteolysis [56] and the phenomenon of “TNF-sinking” in obese patients, which increases the level of TNF inhibitors of fat secretion TNF inhibitors [57]. Therefore, obesity/overweight is also a potential risk factor for increased infectious complications.

In our study, among noninfectious complications, obese IBD patients had an increased risk of visceral injury. Many problems such as obesity-induced changes in abdominal contour and increased abdominal wall thickness pose a greater challenge to the patient’s surgery [49]. Bleeding, complicated surgery, and prolonged surgery are more common conditions [16, 22], which can subsequently lead to visceral injury. Notably, it has been reported in previous studies that among other diseases, such as cancer [58], obstructive sleep apnea (OSA), and obesity hypoventilation syndrome (OHS) [30], obesity was frequently associated with complications such as VTE, adverse cardiovascular events, and unplanned intubation [30]. However, in IBD patients, obesity/overweight is not a risk factor for lifting the occurrence of these complications. This is related to the rejuvenation of IBD patients. Unlike cancer, which is highly prevalent in the middle-aged and elderly population, the population incidence of IBD tends to be younger. Young people have fewer underlying diseases, relatively sound cardiovascular, pulmonary, and other organ functions, as well as coagulation-anticoagulation system, and perfect physical immune function, which give rise to the relatively stable incidence of noninfectious surgical complications in the increasing BMI.

Early in the development of laparoscopy, contraindications for this technique included patients with higher BMI and IBD [11]. However, in recent years, with the development of technology, more and more laparoscopic bowel resection has taken over the traditional laparotomy, and its indications in obese IBD patients are also developing [30]. Many studies have shown that there is no significant difference between obese and nonobese patients in the conversion toward open surgery [15, 59]. In some cases, laparoscopic surgery in obese patients even uses the same criteria and indications as nonobese patients [30]. But according to our study, the risk of IBD patients experiencing conversion complications is higher in both obese and overweight patients. This is in line with the conclusions drawn by Krane Senagore et al. [10, 60]. In fact, laparoscopic surgery in obese patients with IBD is particularly challenging. First of all, the lesion sites are more easily adherent and difficult to expose due to more adipose tissue [10]. Second of all, the use of concomitant steroids and biological immunosuppressive drugs for the treatment of IBD may result in shortening and weakening of the mesentery [11].

There are breakthroughs in this study: Firstly, the sample size of this study is large compared to other meta-analyses, and it is an updated meta-analysis. Secondly, to further investigate the effect of surgical complications in different BMI ranges, we included not only obese IBD patients but also overweight. Last but not the least, complications were classified in more detail.

At the same time, our study also has some shortcomings to emphasize. The studies included in the meta-analysis were all observational studies with bias and heterogeneity more or less, which inevitably reduced the reliability of this study. A random-effects model was therefore chosen to improve confidence. Due to the limitation that the study subjects are almost from medical institutions in the USA, our study conclusions cannot be directly applied to other countries or regions. In addition, BMI itself has some limitations. It has been reported that BMI has a poor linear relationship with total body fat [61]. Hence, it may not be the best measurement tool to reflect parameters of the degree of body adiposity. Due to lack of data, this study could not be analyzed based on other parameters such as waist circumference, waist-to-hip ratio, subcutaneous fat, visceral fat, and the subcutaneous-to-visceral fat ratio [17, 62]. To clarify the complex interplay between obesity/overweight, IBD, and surgical outcomes, further studies on visceral fat or fat distribution could be considered in the future.

In our study, we suggest that obesity/overweight is a risk factor for more complications after surgical treatment in patients with IBD, which mainly are increased risk of infectious complications, wound complications, as well as conversion rate. Given these findings, in order to minimize the risk of surgical complications, obese/overweight patients could control their weight through reasonable perioperative management on the one hand. On the other hand, selecting the appropriate surgical approach, paying attention to the occurrence of infection, and protecting the incision site are also worth considering.

## Supplementary information

Below is the link to the electronic supplementary material.

Supplementary file1 (TIF 306 KB)

Supplementary file2 (TIF 317 KB)

Supplementary file3 (DOCX 18 KB)

## Data Availability

The datasets supporting the conclusions of this article are included within the article and its additional files.
