# Supplementary figures and images for: Systematic review and meta-analysis: association between obesity/overweight and surgical complications in IBD
Source: Int J Colorectal Dis. 2022 May 31;37(7):1485–96. doi: 10.1007/s00384-022-04190-y (PMC9262757; doi:10.1007/s00384-022-04190-y)

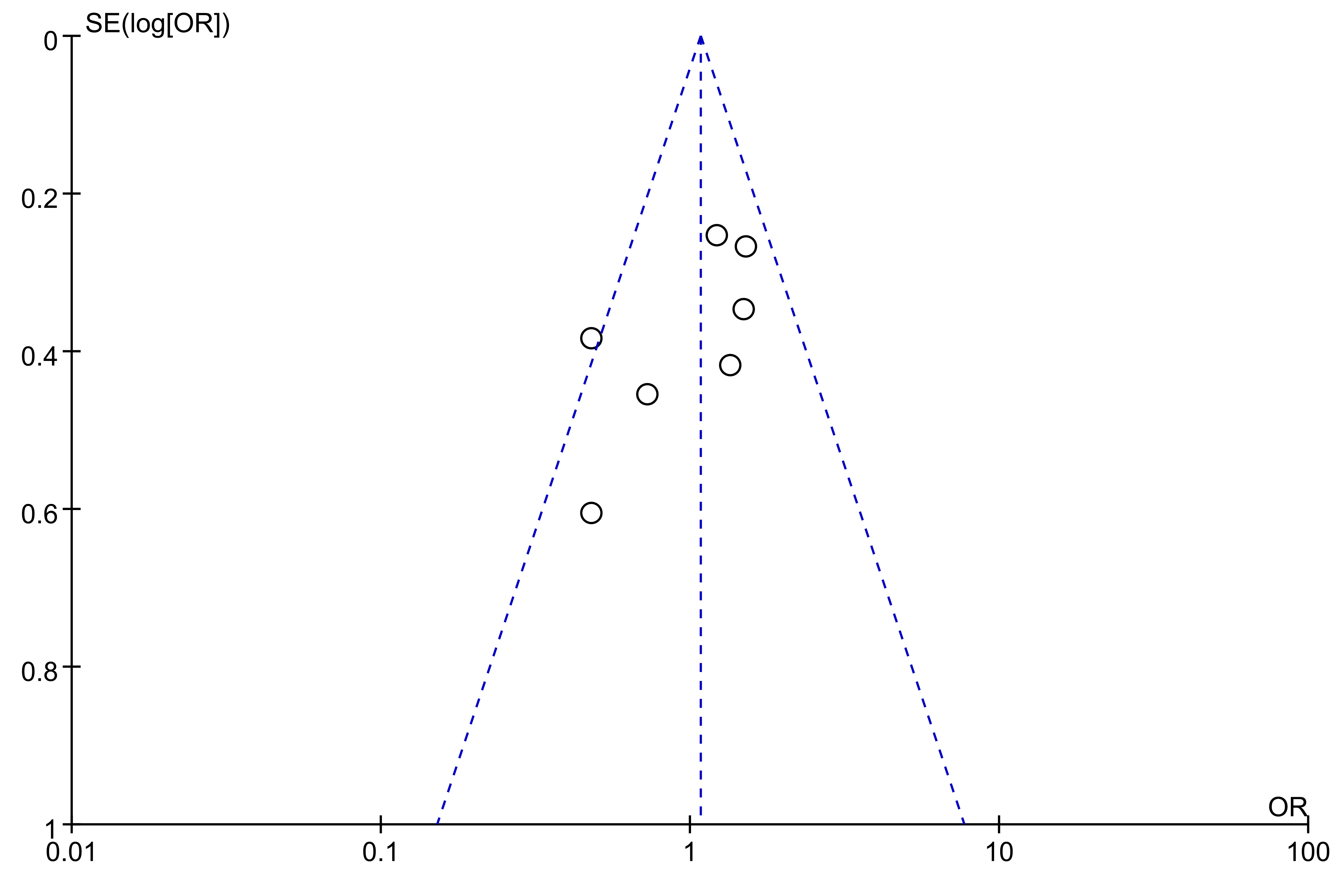

Supplement: Supplementary file 1 — Supplementary file1 (TIF 306 KB) [file 384_2022_4190_MOESM1_ESM.tif]

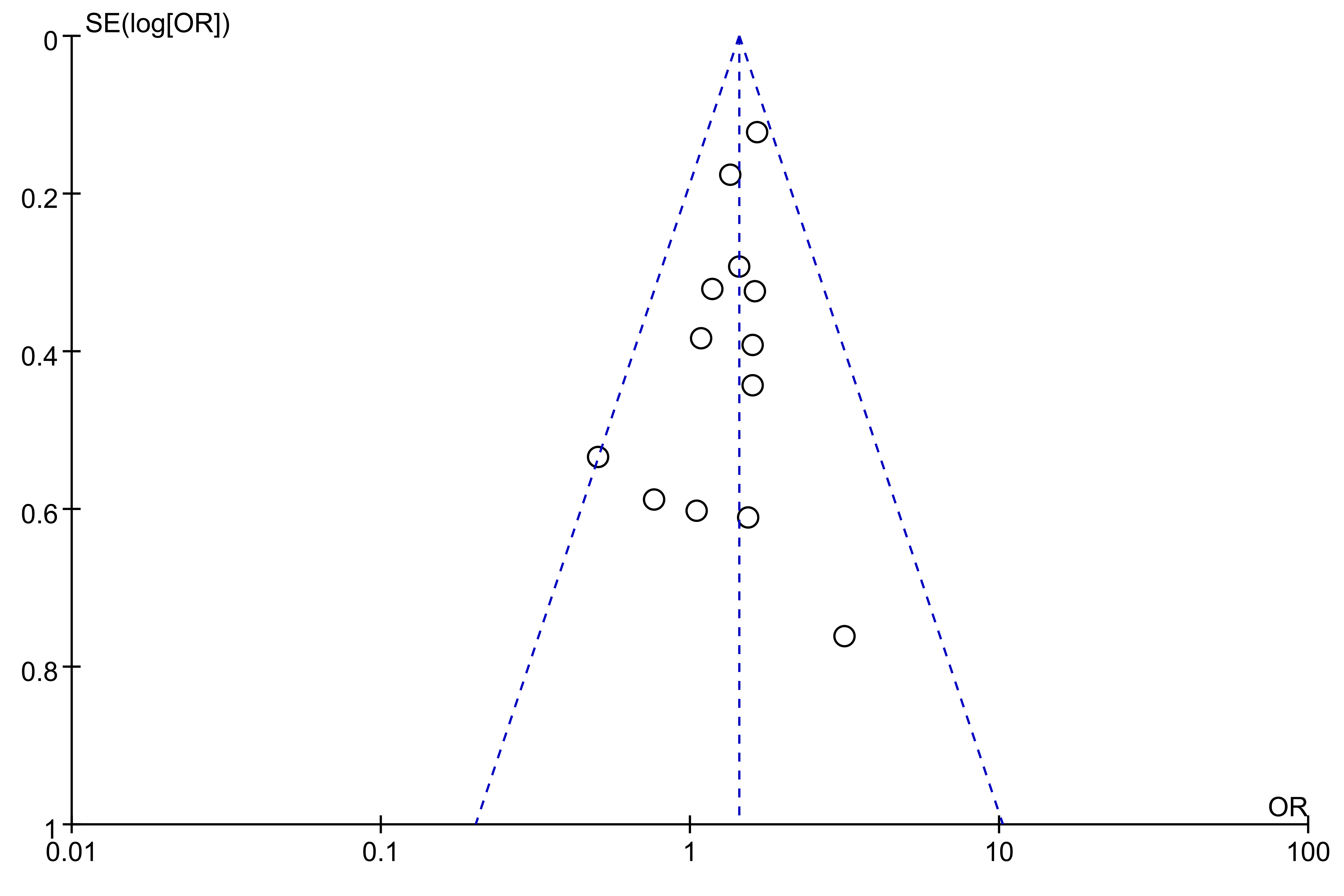

Supplement: Supplementary file 2 — Supplementary file2 (TIF 317 KB) [file 384_2022_4190_MOESM2_ESM.tif]
