# Supplementary material for: Systematic review and meta-analysis: association between obesity/overweight and surgical complications in IBD
Source: Int J Colorectal Dis. 2022 May 31;37(7):1485–96. doi: 10.1007/s00384-022-04190-y (PMC9262757; doi:10.1007/s00384-022-04190-y)
Supplement: Supplementary file 3 — Supplementary file3 (DOCX 18 KB) [file 384_2022_4190_MOESM3_ESM.docx]

**Supplementary Table 1. Quality assessment of studies included.**

| **Autheor,year,**  **Study** | **Selection (Out of 4)** | | | | **Comparability**  **(Out of 2)** | **Outcomes(Out of 3)** | | | **Total**  **(Out of 9)** |
| --- | --- | --- | --- | --- | --- | --- | --- | --- | --- |
|  | Representativeness of exposed cohort | Selection of  nonexposed cohort | Ascertainment  of exposure | Outcome not present at the start of the study |  | Assessment of outcomes | Length of follow-up | Adequacy of follow-up |  |
| Canedo, 2010 | 1 | 0 | 1 | 1 | 2 | 1 | 1 | 1 | 8 |
| Causey, 2011 | 1 | 1 | 1 | 1 | 2 | 1 | 1 | 1 | 9 |
| Krane, 2013 | 1 | 1 | 1 | 1 | 2 | 1 | 1 | 0 | 8 |
| Stidham, 2015 | 1 | 1 | 1 | 1 | 1 | 1 | 1 | 1 | 8 |
| Sahami, 2016 | 1 | 1 | 1 | 1 | 1 | 1 | 1 | 1 | 8 |
| Guardado, 2016 | 1 | 1 | 1 | 1 | 2 | 1 | 1 | 1 | 9 |
| Manne, 2015 | 1 | 1 | 1 | 1 | 1 | 1 | 1 | 0 | 7 |
| Okita, 2017 | 1 | 0 | 1 | 1 | 1 | 1 | 1 | 1 | 7 |
| McKenna, 2017 | 1 | 1 | 1 | 1 | 1 | 1 | 1 | 1 | 9 |
| Heimann, 2018 | 1 | 1 | 1 | 1 | 1 | 1 | 1 | 1 | 8 |
| McKenna, 2018 | 1 | 1 | 1 | 1 | 1 | 1 | 1 | 1 | 8 |
| Horio, 2018 | 1 | 0 | 1 | 1 | 2 | 1 | 1 | 1 | 8 |
| Kao, 2019 | 1 | 1 | 1 | 1 | 1 | 1 | 1 | 1 | 8 |
| McKenna, 2019 | 1 | 1 | 1 | 1 | 2 | 1 | 1 | 1 | 9 |
| Abd EI Aziz, 2021 | 1 | 1 | 1 | 1 | 2 | 1 | 1 | 1 | 9 |

The observational studies were assessed by the Newcastle-Ottawa Quality Assessment Scale.
